# HPV16 E6 gene polymorphisms and the functions of the mutation site in cervical cancer among Uygur ethnic and Han nationality women in Xinjiang, China

**DOI:** 10.1186/s12935-022-02506-0

**Published:** 2022-02-22

**Authors:** Huizhen Xin, Zhenzhen Pan, Xiangyi Zhe, Chunhe Zhang, Hongtao Li, Weinan Zheng, Haichen Long, Renfu Shao, Dongmei Li, Zemin Pan

**Affiliations:** 1 Xinjiang Endemic and Ethnic Disease and Education Ministry Key Laboratory, Department of Biochemistry and Molecular Biology, School of Medicine, Shihezi University, Shihezi, 832002 Xinjiang China; 2 Department of Clinical Laboratory, Xinjiang Production and Construction Corps of the Fourth Division Hospital, Yining, 835000 Xinjiang China; 3 Department of Human Anatomy, Chengdu Medical College, Chengdu, 610000 Sichuan China; 4 School of Science and Engineering, Genecology Research Centre, The Animal Research Centre, University of the Sunshine Coast, Sippy Downs, QLD 4556 Australia; 5 Department of Biology, School of Basic Medical Sciences, Xinjiang Medical University, Urumqi, 830017 Xinjiang China

**Keywords:** HPV genotype, Sequencing analysis, HPV16 E6, Mutation, Cervical cancer

## Abstract

**Background:**

To investigate the genotype distribution of human papillomavirus (HPV) in infected Uygur and Han women in Xinjiang, China; analyze the HPV16 E6 gene polymorphism site and relationship with the development of cervical cancer.

**Methods:**

The HPV16 E6 sequence was analyzed using the European standard prototype to perform an evolutionary tree. HPV16 E6-T295/T350, G295/G350, and T295/G350 GV230 vectors were stably transfected into cervical cancer C33A cells to analyze the cell proliferation, migration and invasion, apoptosis by CCK8 and clonogenic assays, transwell and cell scratch assays, FACS experiments.

**Results:**

The total HPV infection rate was 26.390% (760/2879), whereas the Uygur 22.87% (196/857) and the Han was 27.89% (564/2022) (P < 0.05). Among 110 mutations, 65 cases of E6 genes were mutated at nucleotide 350 (T350G) with the leucine changing to valine (L83V). Moreover, there were 7 cases of E6 gene mutated at nucleotide 295 (T295G) with aspartic changing to glutamic (D64E). When E6 vector(s) of mutations sites were transfected into C33A cells, they were found to promote cellular proliferation, migration, invasion, and inhibit apoptosis. T295/G350-E6 was significantly stronger than G295/G350 and T295/T350, G295/G350 was significantly stronger than T295/T350 (P < 0.05). The T295/G350 had the strongest effect on C33A cells and G295/G350 was significantly stronger than T295/T350 (P < 0.05).

**Conclusions:**

The positive HPV infection rates differed between the Uygur and Han in Xinjiang, China, and the genotype distribution of infection was different. After transfecting C33A cells with different eukaryotic expression vectors, the T295/G350 mutation site promoted the proliferation, migration, and invasion of C33A cells to a greater extent than G295/G350; however, G295/G350 had a stronger effect than T295/T350.

**Supplementary Information:**

The online version contains supplementary material available at 10.1186/s12935-022-02506-0.

## Background

Cervical cancer is a common malignant tumor in women around the world and represents the leading cause of cancer-related death. The incidence and mortality of cervical cancer in women from low-income countries are higher than in high-income countries, and approximately 80–90% of cervical cancer deaths occur in developing countries [1]. Nationally, Xinjiang has both a high prevalence and mortality rate of cervical cancer, and the prevalence and mortality of Han and other ethnic minorities are lower compared to that of Uyghurs.

Many factors are associated with the occurrence and development of cervical cancer. German scientists first discovered that human papillomavirus can cause cervical cancer [2]. More than 20 of the detected HPV types are classified as high-risk HPV, of which HPV16 is most closely related to cervical cancer. The two most common high-risk types are HPV16 and HPV18, which cause about 70% of all cervical cancer. Moreover, while most women will have at least one type of HPV infection in their lifetime, the majority of these infections are subclinical and are associated with self-limiting conditions. HPV is usually cleared by the body's immune system within 6–18 months. Only about 10% of persistent viral infections will experience long-term progression from low-grade to high-grade cervical intraepithelial neoplasia (CIN) and eventually develop into cervical cancer [3–5]. In addition, HPV alone is not highly carcinogenic, and most HPV infections are undetectable after a few months and do not cause cancer. In addition, about 2% of infections cannot be detected after two years. There may also be persistent latent infections that cannot be detected using PCR methods due to low viral loads [6]. In addition, cervical basal cell differentiation is a signal for HPV to transition from a latent infection to a productive infection [2]. During this process, the host cell genome integrates with the HPV genome and releases the progeny virus, resulting in a persistent HPV infection. The expression of a viral oncogene leads to changes in the expression of host cell-related genes. Accordingly, an abnormal number or structure of host chromosomes occurs and eventually develops into cervical cancer [7]. HPV is a small double-stranded DNA virus and its persistent infection is associated with cervical cancer and other anal, genital, and head and neck cancers. Alpha-type high-risk HPV (hrHPV) (HPV 16, 18, 31, 33, 35, 39, 45, 51, 52, 56, 58, 59 and 68), is often found in cervical cancer [8]. Although HPV16 is the most common high-risk HPV responsible for causing cervical cancer, the mechanism by which it promotes cervical cancer is under investigation.

Epidemiological studies have confirmed that different HPV16 variants exhibit variable carcinogenic potential and biological activity, and also displayed regionality in relation to host HLA polymorphisms [9–11]. Moreover, different HPV16 subtypes have distinct carcinogenic potential and may be associated with mutations in certain important sequence sites [12–15]. Some studies have found that HPV16 shows an extremely high infection rate in Xinjiang Uygur cervical squamous cell carcinoma, with a detection rate of 96.7% [14]. In addition, the infection rate of HPV16 in cervical cancer tissues in Xinjiang Uygur women was previously detected to be 69.23% [15]. HPV16 carcinogenesis is primarily related to the activity of oncogenes, E6 and E7, which can bind to some cellular proteins, such as p53 and pRb, thereby interfering with the apoptosis, differentiation, adhesion, cell cycle, and immune response of normal cells to promote carcinogenesis of the cervix [16, 17]. Studies have shown that mutations at multiple sites in the E6 gene can affect the antigenicity, immunogenicity, and susceptibility of the HPV16 virus [18, 19]. T350G is the most common polymorphic mutation in European strains, which causes leucine to change to proline (L83V). T178G is the most common polymorphic mutation in Asian strains, which causes aspartate to change to glutamine (D25E) [20]. Therefore, it is important to understand the sequence characteristics of HPV16 mutations in this region.

In the same environment, the incidence of cervical cancer is higher in Xinjiang Uygur women compared to those of Han nationality. This difference may be attributed to personal factors such as hygiene and living habits, or it could also indicate a differential molecular evolution of the HPV16 virus. In this study, HPV epidemiological data were used to analyze the distribution of HPV genotypes among infected women of Xinjiang Uygur and Han nationality. The HPV16 E6 gene was amplified by PCR and sequenced. After sequencing, the evolution of the HPV16 E6 gene was analyzed using MEGA6.0 bioinformatics software to assess the relationship between the molecular evolution of the high-risk HPV16 E6 gene and the occurrence and development of cervical cancer in Xinjiang Uygur and Han women. The location of the HPV16 E6 gene-specific pathogenic polymorphism site and the role of polymorphic sites in the development and progression of cervical cancer was investigated using eukaryotic transfection experiments. This information helps people fully understand HPV and provides novel insight into the diagnosis and treatment of HPV-induced cervical cancer. These findings may provide a basis for HPV vaccine development, which lays a foundation for the prevention and treatment of cervical cancer.

## Methods and materials

### Sample collection

Samples of exfoliated cells from the cervix of Uygur and Han women after HPV typing collected from 2015 to 2017 at Yili Friendship Hospital in Xinjiang, China as well as the clinical data of patients were included in this study. The inclusion criteria consisted of women who were sexually active and had an intact cervix. The HPV genotyping results of the exfoliated cells consisted of 2879 cases, including 207 HPV16-positive samples that were retrospectively analyzed. Samples of the cervical tissue of Uyghur women were collected from 2011 to 2014 at the Kashgar Hospital and People’s Hospital of the Xinjiang Uygur Autonomous Region. A total of 72 cases of HPV16 virus-infected cervical samples were screened by HPV16 E6 primers. The samples were frozen at − 80℃.

### Bioinformatics analysis and construction of an evolutionary tree

The HPV-infected samples were collected and sequenced by the Shanghai Tianhao Biotechnology Company. The PCR products were purified using SAP (Promega) and Exo I (Epicentre) and sequenced using the ABI Big-Dye Terminator v3.1 Cycle Sequencing Kit and the ABI3130XL Genetic Analyzer. The sequencing results were analyzed using Polyphred software for single nucleotide polymorphisms (SNP), and compared to the HPV16 European standard strain (NC_001526.1) and other HPV16 variants, the phylogenetic tree was constructed by software MEGA 6.0. In this study, the partial sequence for the HPV16 E6 gene was identified on the NCBI website GenBank (Accession Number E6: KT959524–KT959566).

### Cell culture

The C33A cell strain was prepared in the laboratory, and the HPV16 E6 plasmid was purchased from Addgene, USA. Subsequently, these cell lines were cultured in HIGH-DEME (Gibco, America) medium supplemented with 10% fetal bovine serum (FBS, SiJiQing, China), 100 U/mL penicillin, 50 μg/mL streptomycin, and 800 μg G418 for cells transfected with HPV plasmid in incubators under a humidified atmosphere of 5% CO_2_ and 95% air at 37 °C.

### Plasmid construction and transfection

The GV230 vector had an element sequence of: CMV-MCS-EGFP-SV40-Neomycin and the cloning site in XhoI/KpnI, HPV16 E6 plasmid map is presented in Additional file 1: Fig. S1. The primer was designed and synthesized by Shanghai Jikai Gene Company. The primer information is presented in Table 1. The C33A cells were inoculated into six well plates, and the HPV16 E6 plasmid was transfected using an FuGENE HD transfection reagent (Promega, America) according to the manufacturer’s instructions. After 48 h of transfection, the HPV16 E6 protein was successfully detected by indirect immunofluorescence and the morphological changes in the C33A cells stably transfected with HPV16 E6 were also observed by transmission electron microscopy. HPV16 E6 antigen was purchased from Santa Reagent Company, USA. Afterwards, stably growing cells containing the target gene were collected to confirm the transfection efficiency or further functional assays.

**Table 1:** HPV16 E6 primer information

Gene	Sequence
HPV16 E6 prototype-P1	TACCGGACTCAGATCTCGAGCGCCACCATGCACCAAAAGAGAACTGCAATG
HPV16 E6 prototype-P2	GATCCCGGGCCCGCGGTACCGTCAGCTGGGTTTCTCTACGTGTTC
HPV16 E6-G350 mutation-P1	TACCGGACTCAGATCTCGAGCGCCACCATGCACCAAAAGAGAACTGCAATG
HPV16 E6-G350 mutation-P2	GATCCCGGGCCCGCGGTACCGTCAGCTGGGTTTCTCTACGTGTTC
HPV16 E6-G295/G350 mutation-P1	TACCGGACTCAGATCTCGAGCGCCACCATGCACCAAAAGAGAACTGCAATG
HPV16 E6-G295/G350 mutation-P2	GATCCCGGGCCCGCGGTACCGTCAGCTGGGTTTCTCTACGTGTTC

### Cellular proliferation assay

At 48 h post-transfection of the four aforementioned vectors, the cells were evenly spread into 96-well plates at a concentration of 3000 cells per well, and the relative number of viable cells was measured with Cell Counting Kit-8 reagents (Dojindo Laboratories, Kyushu Island, Japan) at 24, 48, and 72 h, respectively. The absorbance at 450 nm was used to reflect the results using a microplate reader (Bio-Rad, USA).

### Colony formation assay

C33A cells were seeded and transfected with an HPV16 E6 plasmid on a six-well plate. After 24 h, the transfected cells were seeded into another six-well plate at a concentration of 1000 cells per well and cultured for another 14 days. The cells were then fixed with methanol for 15 min and then stained with 0.1% crystal violet for 15 min. The colonies were counted under the low power of the microscope manually for greater than 50 cell clones, and each experiment was performed three times independently.

### Transwell assay

The transwell migration assay was performed in a Transwell chemotaxis 24-well chamber (Corning, American). A density of 3 × 10^4^ cells in 200 μL serum-free medium were plated in the upper chambers, which is collected after stably transfecting each plasmid. 600 µL of complete medium containing 20% FBS was added to the lower chambers. 600 µL of 4% paraformaldehyde (Boster Biological Technology, Wuhan, China) was used for room temperature fixation, and 600 µL of 0.1% crystal violet (Solarbio, Beijing, China) was used for staining. For the invasion assay, the basement membrane of the filters was coated with 20 μL Matrigel (Corning, American). After incubating the cells for 48 h, the cells that had migrated or invaded the lower surface of the membrane were stained with crystal violet. The fixation and staining process is the same as the migration experiment. The results were all determined by counting the stained cells using optical microscopy (200× magnification) in five randomly selected fields. Each experiment was carried out in triplicate wells and repeated at least three times.

### Apoptosis evaluation

The cells were digested with trypsin without EDTA (Solarbio, Beijing, China), and then 2 × 10^6^ to 5 × 10^6^ cells were collected for each experimental group. After washing twice with 1× PBS and centrifuging, the cells were resuspended in 500 μL of pre-cooled 1× Binding Buffer to a cell concentration of 1 × 10^6^ to 1 × 10^7^ cells/mL. Next, 100 μL of the cell suspension was added to each sample tube, with 5 μL Annexin V-APC and 10 μL 7-AAD. The suspension was gently mixed and incubated for 15 min at room temperature in the dark. Next, 380 μL of pre-cooled 1 × Binding Buffer was added to each tube and mixed before flow cytometry testing.

### Nude mouse xenograft model experiment

The C33A cervical cancer cells stably transfected with the recombinant vector were digested, and 200 μL of the cell suspension, of which the concentration was 1.5 × 10^7^/mL, was slowly and subcutaneously injected into the left hind leg of the 4–6-week-old nude mice (purchased from Viton Lihua, Laboratory Animal Co., Ltd., Beijing, China, one week after adaptive feeding in an SPF-class conditional animal experiment center). The nude mice were housed for 4 weeks, during which, the weight of each group of nude mice was weighed every three days, and the size of the tumor was measured with a Vernier caliper. The volume of the tumor was calculated using the following formula: volume = π/6 × long diameter × square of the short diameter. After 4 weeks, the nude mice were sacrificed and the tumors were removed for subsequent experiments. The method of killing nude mice uses the neck-breaking method, which is the most common method of killing mice and the method of minimizing the pain of mice, which is in accordance with animal ethics.

### Statistical analysis

SPSS19.0 statistical software was used to analyze and process the data. At least three independent repeats were performed for each experiment. All values are expressed as the mean ± standard deviation. A threshold of P < 0.05 was considered statistically significant.

## Results

### HPV16 E6 gene sequencing and polymorphism analysis

**Fig. 1:**
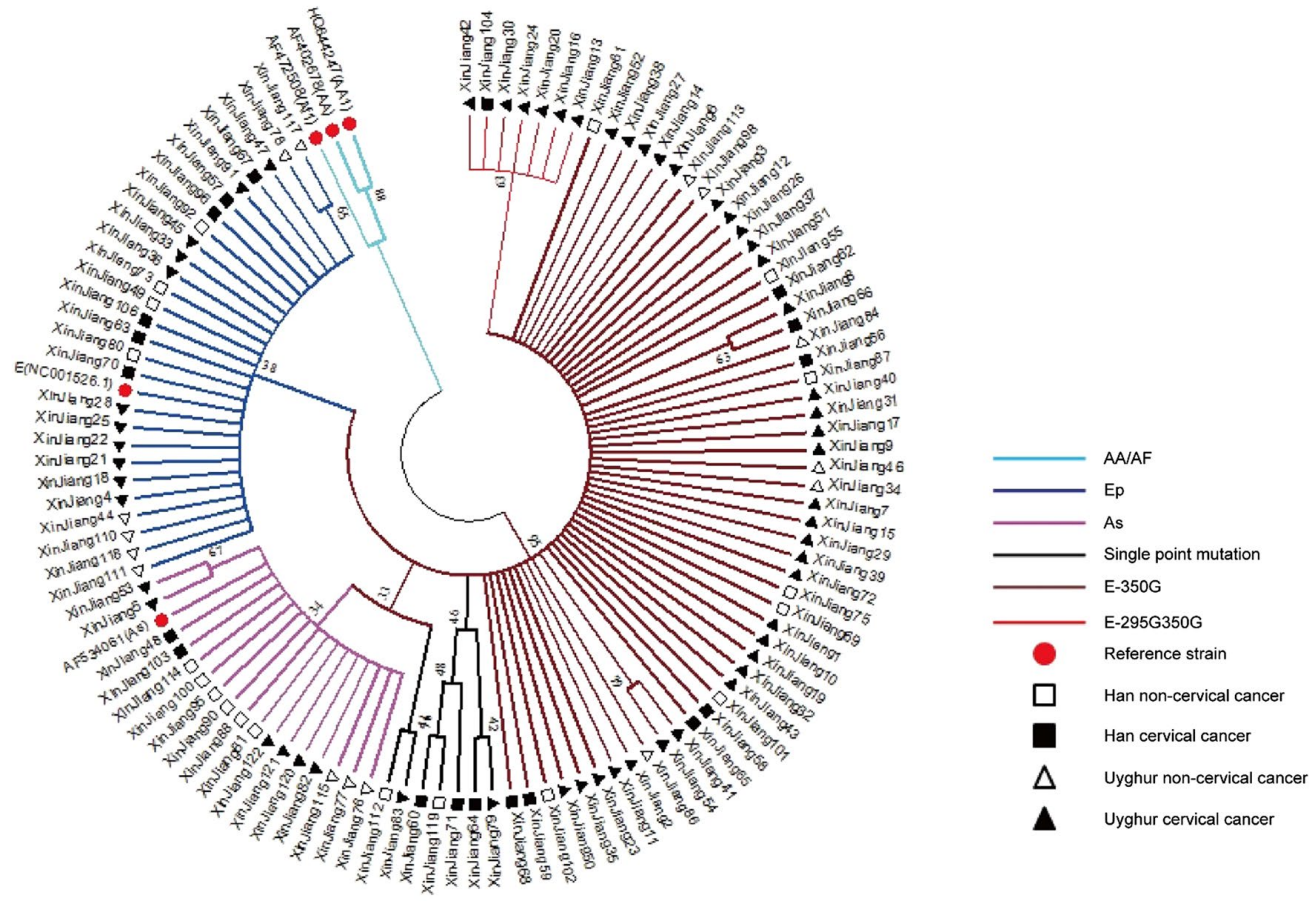
Phylogenetic tree analysis of the HPV16 E6 gene

The full-length sequence of the HPV16 E6 gene was determined in 110 HPV16-positive patients. A total of 14 mutation sites were identified, including six synonymous mutations and eight missense mutations. The genetic variants of the sequences in comparison to the European standard strains are shown in the Additional file 1: Tables S1–S4. The phylogenetic tree analysis revealed that among the 110 samples, 27 belonged to the HPV16 European type (Ep), 17 belonged to the Asian strain (As), 59 belonged to European variant (E), and 7 of European variants became an independent branch, related to the E6 T295G mutation. Another seven cases displayed only a single point mutation (Fig. 1).

### Expression of HPV16 E6 in cervical cancer C33A cells

A GV230 empty vector, HPV16 E6-T295/T350 prototype vector, HPV16 E6-G295/G350 mutation vector, and HPV16 E6-T295/G350 mutation vector were transfected into cervical cancer C33A cells, and the expression of HPV16 E6 was observed with a microscope after 48 h. As shown in the Additional file 1: Fig. S2, the level of HPV16 E6 protein expression was detected by indirect immunofluorescence using an HPV16 E6 antibody. The GV230 empty vector group was used as a control group and did not exhibit any red fluorescence, which indicated that the C33A cells did not express the HPV16 E6 protein; however, red fluorescence was observed in the other three experimental groups.

### Effect of an HPV16 E6 mutation on C33A cell proliferation

**Fig. 2:**
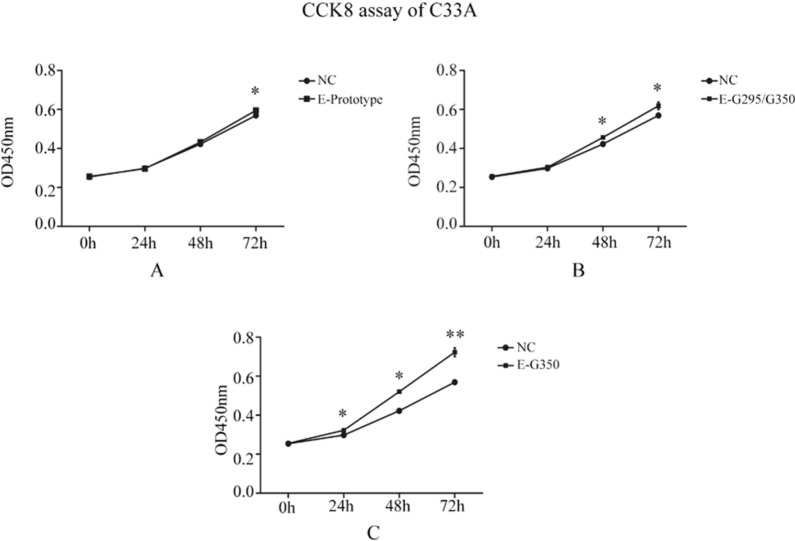
The effect of an HPV16 E6 mutation on the proliferation of C33A cells. **A** The proliferation of the HPV16 E6 prototype group at different times compared to the empty vector NC group. **B** The proliferation of the HPV16 E6-G295/G350 mutant group at different times compared with the empty vector NC group. **C** The proliferation of the HPV16 E6-G350 mutant group at different times compared with the empty vector NC group. *P < 0.05; **P < 0.01 were considered to be statistically significant

As shown in the Fig. 2, there was a significantly different optical density (OD) between the HPV16 E6 prototype group and the NC group at 72 h (P < 0.05). There was also a significant difference in the OD between the HPV16 E6-G295/G350 mutation group and the NC group at 48 h and 72 h (P < 0.05). There was a significant difference in the OD of the HPV16 E6-T295/G350 mutation group and NC group at both 24 h and 48 h (P < 0.05). In addition, the OD of the HPV16 E6-T295/G350 mutation group and NC group were significantly different at the 72 h time point (P < 0.01) (Fig. 2).These results suggest that the cells in the experimental group that express HPV16 E6 have a significantly higher proliferation capacity compared to that of the control group. Among three experimental groups, C33A cells have the strongest proliferation ability in the presence of an HPV16 E6-T295/G350 mutation.

### Effect of HPV16 E6 mutation on C33A cell clone formation

**Fig. 3:**
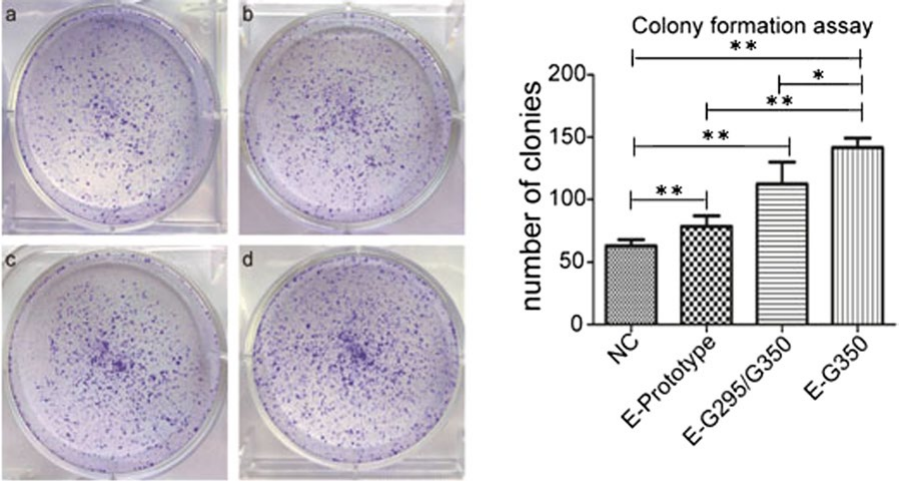
Effect of an HPV16 E6 mutation on the formation of C33A cell clones. **a** GV230-transfected NC group; **b** HPV16 E6 prototype group; **c** HPV16 E6-G295/G350 mutation group; d. HPV16 E6-T295/G350 mutation group. *P < 0.05; **P < 0.01 were considered statistically significant

As shown in Fig. 3 the results of this experiment are consistent with the CCK8 proliferation experiments. The number of colonies in the three experimental groups was significantly higher than that in the NC control group. The HPV16 E6-T295/G350 mutation had the greatest effect on the formation of C33A cells. Moreover, there were significant differences between the NC group and each HPV16 E6 expression group (P < 0.01).

### Effect of HPV16 E6 mutation on the migration and invasion of C33A cells

**Fig. 4:**
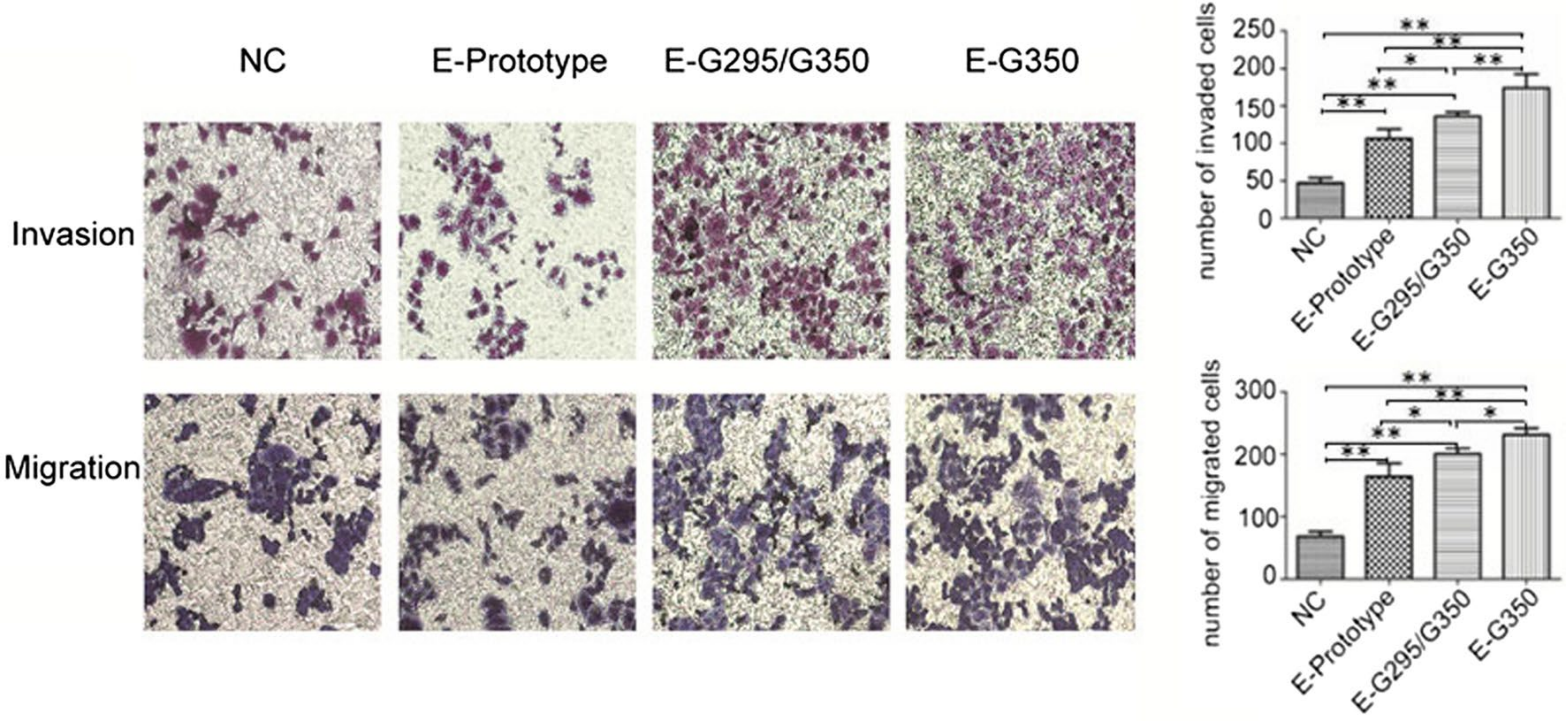
The effect of an HPV16 E6 mutation on the migration and invasion of C33A cells. After C33A cells were transfected with a GV230 empty vector, HPV16 E6 prototype vector, HPV16 E6-G295/G350 mutation vector, and HPV16 E6-G350 mutation vector, a transwell invasion assay and migration assay of the C33A cells were performed. *P < 0.05; **P < 0.01 were considered statistically significant

The transwell invasion assay results showed that there were significantly fewer cells in the NC group compared to the HPV16 E6 prototype, HPV16 E6-G295/G350 mutation, and HPV16 E6-T295/G350 mutation groups (P < 0.01). There were fewer cells in the HPV16 E6 prototype group compared to that in the HPV16 E6-G295/G350 mutation group (P < 0.05) and the HPV16 E6-T295/G350 mutation group (P < 0.01). There were fewer cells in the HPV16 E6-G295/G350 mutation group compared to that in the HPV16 E6-T295/G350 mutation group (P < 0.01) (Fig. 4). The transwell migration assay results showed that following the stable transfection of cervical cancer C33A cells, the cells in the GV230 empty vector control group (NC) displayed significantly less permeation than that of the HPV16 E6 prototype, HPV16 E6-G295/G350 mutation, and HPV16 E6-T295/G350 mutation groups (P < 0.01). The HPV16 E6 prototype group (E6-T295/T350) had fewer perforating cells compared to the HPV16 E6-G295/G350 mutation group (P < 0.05) and HPV16 E6-T295/G350 mutation group (P < 0.01). The HPV16 E6-G295/G350 mutant group had fewer perforating cells than the HPV16 E6-T295/G350 mutation group (P < 0.05) (Fig. 4). These results indicate that there was a significant difference between the HPV16 E6 expression group and the NC group (P < 0.05). In addition, the expression of the HPV16 E6-T295/G350 mutation had the greatest promoting effect on the migration and invasion of C33A cells.

### Effect of HPV16 E6 mutations on the apoptosis of C33A cells

**Fig. 5:**
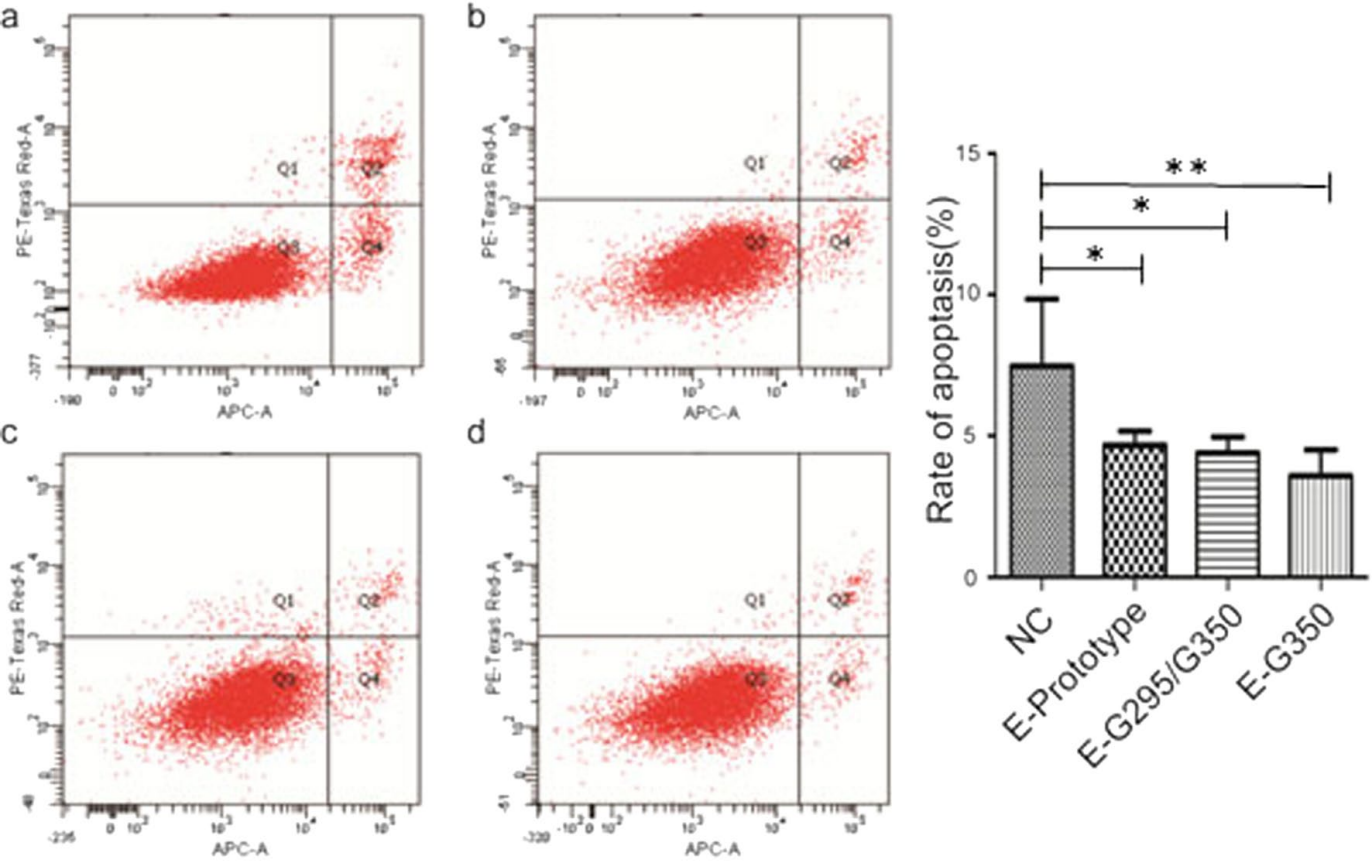
The effect of an HPV16 E6 mutation on the apoptosis of C33A cells. **a** GV230 transfected NC group; **b** HPV16 E6 prototype group; **c** HPV16 E6-G295/G350 mutation group; d. HPV16 E6-G350 mutation group. *P < 0.05; **P < 0.01 were considered statistically significant

The apoptosis rate of the cells in each group was detected by flow cytometry. Among the cervical cancer C33A cells, the apoptosis rate of the cells in the NC group was significantly higher than that of the HPV16 E6 prototype group (P < 0.05), HPV16 E6-G295/G350 mutation (P < 0.05), and HPV16 E6-T295/G350 mutation groups (P < 0.01). There were differences observed among the HPV16 E6 prototypes, HPV16 E6-G295/G350 mutation, and HPV16 E6-T295/G350 mutation in these three experimental groups; however, these differences were not statistically significant (P > 0.05) (Fig. 5). This finding suggests that the HPV16 E6 mutation inhibits cervical cancer C33A cell apoptosis.

### Effect of the HPV16 E6 mutation site on tumor formation in nude mice

**Fig. 6:**
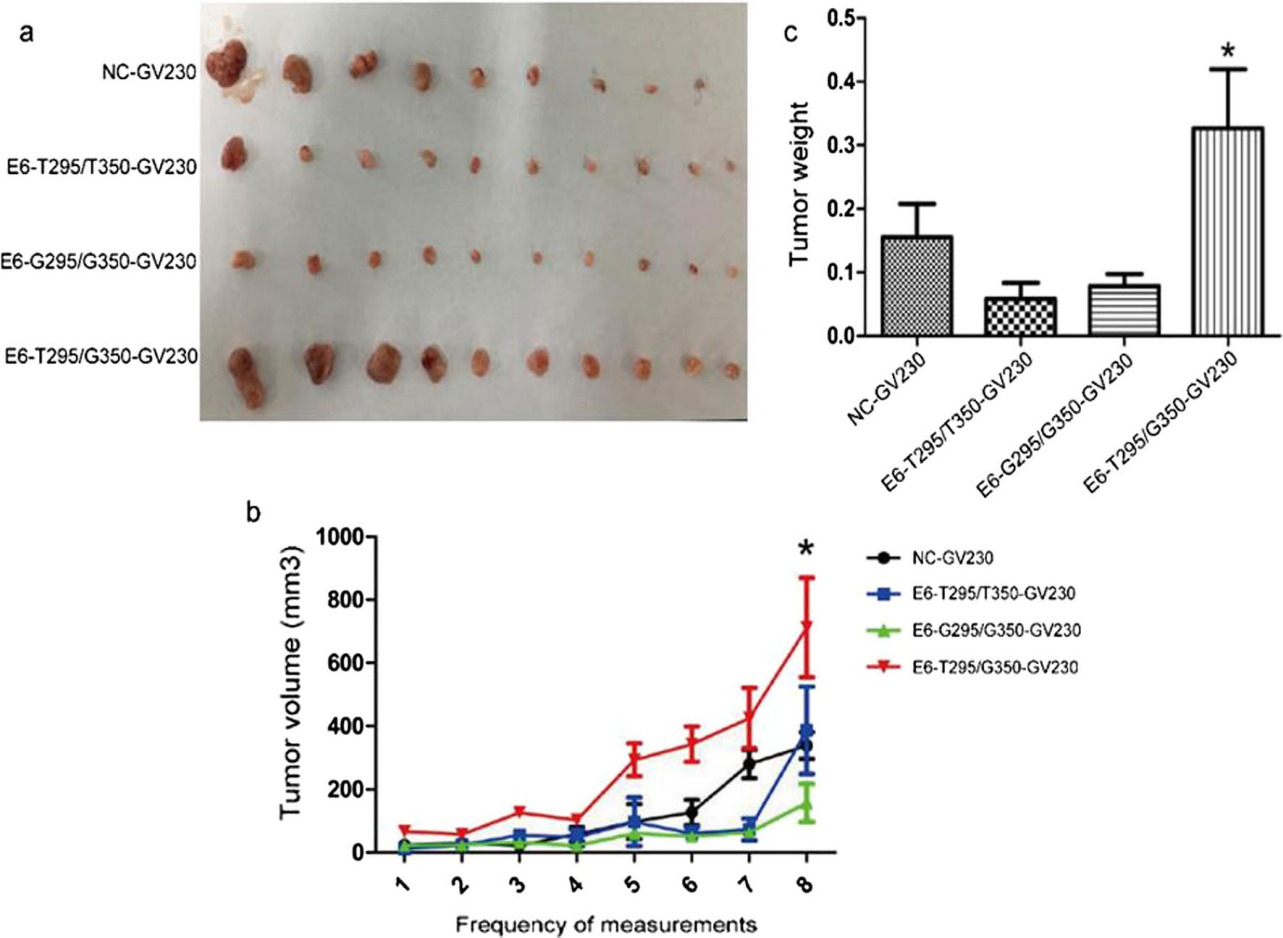
Nude mouse xenograft tumor experiment. **a** Comparison of the final tumor volume of the nude mice after death. **b** Tumor growth curve. **c** The tumor weight of the nude mice after death

After the nude mice were sacrificed, the average terminal tumor volume was found to be 338.77 mm^3^ in the NC group, 264.18 mm^3^ in the E6 prototype group, 157.43 mm^3^ in the E6-G295/G350 mutation group, and 712.98 mm^3^ in the E6-T295/G350 mutation group. There was no significant difference among the HPV16 E6 prototype, E6-G295/G350 mutation, and NC groups (P > 0.05). However, the tumor volume in the HPV16 E6-T295/G350 mutation group was significantly larger than that of the NC group and other experimental groups (P < 0.05) (Fig. 6a and b). The terminal tumor weight is presented in Fig. 6c: the NC group was 0.155 g, E6 prototype group was 0.058 g, E6-G295/G350 mutation group was 0.073 g, and E6-T295/G350 mutation group was 0.297 g. A comparison of the groups using a *t*-test showed that the HPV16 E6-T295/G350 mutation group was significantly increased compared with the NC group (P < 0.05). Together these experimental results showed that an HPV16 E6-T295/G350 mutation can further promote the tumor growth of cervical cancer.

## Discussion

Although HPV infection is associated with the occurrence of a variety of tumors, it is particularly associated with cervical cancer [21]. Multiple studies have confirmed that persistent infection with HPV is the definitive cause of cervical cancer, making HPV screening critically important. Our results show that the positive rate of total HPV infection in females in Yili, the westernmost part in Xinjiang of China, is 26.39%, the positive rate of Han infection is 27.89%, and the positive rate of Uyghur infection is 22.87%. Thus, there was an observed difference in the positive rate of infection between the two ethnic groups. Among the permanent residents of Yili, the Uygur population accounts for approximately 47%, and the Han nationality accounts for about 36%. However, the Uyghur women in the test population accounted for only 20%, which may be related to health awareness and the socioeconomic status. Studies have shown that [22] among HPV-infected women, probability of cervical cancer in multiple parturients is significantly higher than in non-fertile women. In contrast, while the Uygur lifestyle is typically associated with early marriage, early childbearing, multiple births, and intensive births. This study shows that HPV16, HPV52, and HPV53 represented the high-risk HPV strains with a high detection rate in this region, especially in Uygur. The HPV16 infection rate in Uyghur was 25.19%, which was significantly higher than that of the women of Han nationality, at 18.04%. HPV16 was identified to be the most carcinogenic of the high-risk HPV strains, which may be related to the high prevalence of cervical cancer in Uygur. There are also regional differences in the positive rate of HPV infection. Li et al. [23] reported that the positive rate of HPV infection among the Beijing population was 19.1%, of which the most common genotypes were HPV52, HPV16, and HPV58. In addition, Zhang et al. [24] reported a positive rate of infection in the Shenzhen population of 15.9%. The most common genotypes were HPV52, HPV16, and HPV53. Mijit et al. [25] reported that the positive rate of HPV infection was 13% in Kashgar, Xinjiang, China, and the most common high-risk types were HPV16, HPV58, and HPV39. The high-risk HPV infection rate in Uruguay was 20.8%, and the most common high-risk types were HPV16, HPV31, HPV51, HPV56, and HPV39 [26]. Although some differences were observed between the positive infection rate and the most common subtypes, HPV16 was the most common genotype in each study. In the present study, the Han infection types were HPV16, 52, 58, 53, 81, 51 42, 33, 31, 56, 66, 68, 43, 6, 18, 59, 14, 11, 35, 45, 73, 82, and 83. The Uyghur infection types were HPV16, 52, 53, 42, 66, 31, 59, 81, 58, 43, 35, 51, 73, 18, 6, 39, 45, 56, 82, 33, 68, 11, and 83. In terms of multiple infections, single infections were most common in both ethnic groups, followed by a double infection, with triple and higher infections being the most rare. Most women are infected with HPV as a transient infection that can be readily cleared by the immune system. Only about 10% of persistent infections are converted into high-grade CIN by low-grade cervical intraepithelial neoplasia (CIN). This type of infection can eventually evolve into cervical cancer, a process that usually takes 5–10 years. It is important to note that while the total infection rate of Uyghur HPV is lower than that of the Han nationality, the infection rate of HPV16 is higher than that of the Han nationality. Thus, Uyghur women may have a decreased ability to clear an HPV16 infection and continue to remain infected with HPV for longer periods of time.

This observed difference may also be related to changes in the body’s hormone levels or HPV susceptibility in Uyghur women. In addition, the prolonged duration of HPV infection may reduce the function of the immune system [27, 28]. At the molecular level, HPV is a double-stranded DNA virus that encodes six early genes (E1, E2, E4, E5, E6 and E7) and two late genes (L1 and L2). Although both E1 and E2 are involved in genomic DNA replication and transcriptional regulation of the virus, the effective tumorigenicity of HPV is primarily caused by its early genes E5, E6, and E7 [29–32]. Among the HPV subtypes, HPV16 is the most common in cervical cancer and its genome is polymorphic.

Evolutionary analysis shows that the worldwide occurrence of HPV16 genomic polymorphisms have evolved over the past 200,000 years, with the following six phylogenetic branches: European (EUR), Asian (As), Asian-American (AA), African 1 (AF-1), Africa type 2 (Af-2), and North American type (NA). Each branch can be further subdivided into endogenous single nucleotide polymorphisms that can detect different variants in persistently infected precancerous lesions or cancer hosts [33]. Epidemiological investigations have shown that different mutants of the same HPV type have different biological characteristics and may be associated with different risks of disease [34]. Changes in E6 coding can induce strong mechanical and functional changes, resulting in large differences in the cancer risk caused by HPV16 variants [35]. Several studies have reported a high degree of similarity in the distribution of HPV16 subtypes among Chinese Han women, mainly the HPV16 Asian and European strains [36, 37]. Our study observed the genetic diversity and phylogeny of 110 samples by sequencing analysis. Compared with the HPV16 European standard strain, there was a total of 27 European standard strains. A total of 14 mutation sites were identified, including six synonymous mutations and eight missense mutations. In addition, there were 17 cases of E6 gene mutations at 178th nucleotides (T178G) belonging to the Asian standard strain. There were 65 cases of E6 gene mutations at 350 nucleotides (T350G) with a mutation frequency of 63.64%, and corresponding amino acid change of leucine → valine (L83V). This belonged to the European strain mutant, 40 of which were Uygur cervical cancer cases. We believe that HPV16 in Han and Uyghur women in Xinjiang is dominated by the Asian and European strains, and there are no Asian and African strains. These results suggest that the infection and development of invasive cervical cancer in Xinjiang Uygur women may be related to the G mutation in the 350th point of the HPV16 European strain. Studies have shown that HPV16 E6-T350G mutations in Moroccan women are prevalent in high-grade cervical lesions and are closely related to the progression of cervical cancer [38]. In addition, the HPV16 E6-G350 (T295/G350) mutant in HPV16 persistently infected cervical diseases and women with malignant cervical cancer is more common than the HPV16 European original strain, HPV16 E6-T350 (T295/T350) [39, 40]. Our study found seven HPV16 E6 T295G and T350G nucleotide co-mutations, which may serve as a variant of the HPV16 European mutant strain. To further study the function of these mutations, three plasmids of the European strain, HPV16 E6 prototype (T295/T350), HPV16 E6-G295/G350 mutation, and HPV16 E6-G350 mutation (T295/G350) were constructed, and the corresponding cytology was designed to verify their function.

In cytology studies, HPV-negative cervical cancer C33A cells were selected for transfection, and a series of cytological methods were used to analyze the effects of different mutants of the HPV16 E6 oncoprotein gene. C33A cells are cervical cancer cells with mutations in the pRB and p53 genes, which does not have the carcinogenic effects of the high-risk HPV oncoprotein, E6 [41]. Therefore, the effect of the HPV16 E6 gene on this cell line can be studied in this cell model. Each mutation expresses a specific gene, and most of these genes are involved in adhesion, proliferation, apoptosis, migration, and invasion. Compared to the HPV16 E6 European strain prototype (T295/T350) and two mutant types (HPV16 E6-T295/G350 mutation and HPV16 E6-G295/G350 mutation stably transfected C33A cells), the present results showed that the HPV16 E6 prototype, HPV16 E6-G295/G350 mutation, and HPV16 E6-T295/G350 mutation promoted cell proliferation, migration, and invasion, and inhibited apoptosis. The HPV16 E6-T295/G350 mutation had the strongest effect on cell proliferation, migration, and invasion, followed by the HPV16 E6-G295/G350 mutation, and HPV16 E6 prototype with the weakest effect. Thus, our results may partially explain the carcinogenic potential of this mutation.

Some experimental studies have shown that the natural variability of the HPV16 E6 gene mutant is sufficient to alter the cell functional activity induced by E6, including resistance to serum/calcium differentiation, prolongation of the primary human keratinocyte lifespan, and P53 and Bax in human immortalized cells. Decreased expression levels, apoptosis, transformation, and immortalization of human keratinocytes have been demonstrated; however, the mechanisms responsible for these changes and the involvement of genes in their regulation remain unclear [42, 43]. One limitation of this study is that there was no further study of the gene expression regulated by the HPV16 E6-T295/G350 mutation and HPV16 E6-G295/G350 mutation. Thus, such mechanisms will be investigated in future studies. While HPV vaccines are currently used in some developed countries, in underdeveloped countries and regions, including China, the high cost of these vaccines remains an obstacle for their wide-spread use. Therefore, some scholars have proposed a semi-compulsory HPV vaccination program in China [44].

## Conclusion

In summary, this study reports the distribution of the HPV infection subtypes in different ethnic and age groups in the region, which helps people fully understand HPV, provides evidence for HPV prevention and treatment in this region, as well as new diagnostic and therapeutic targets for HPV-related tumors and vaccine development. And the polymorphic sites mentioned above may predict the prognosis of cervical cancer.

## Supplementary Information

**Additional file 1**: **Figure S1.** Plasmid map of HPV16 E6. **Figure S2.** The expression of HPV16 E6 in C33A cells by indirect immunofluorescence (200 ×). Following the transfection of C33A cells with a GV230 empty vector, HPV16 E6 prototype vector, HPV16 E6-G295/G350 mutation vector, HPV16 E6-T295/G350 mutation vector, the red fluorescence of the vector was measured. The GV230 empty vector group was used as a control group and displayed no red fluorescence, whereas red fluorescence was observed in the other three experimental groups. The HPV16 E6-T295/G350 mutation group contained the highest fluorescence. **Table S1.** HPV16 E6 gene mutation loci in the non-cervical cancer of Han women. **Table S2.** HPV16 E6 gene mutation loci in the non-cervical cancer of Uygur women. **Table S3.** HPV16 E6 gene mutation loci in the cervical cancer of Han women. **Table S4.** HPV16 E6 gene mutation loci in the cervical cancer of Uygur women.

## Data Availability

The datasets used and analyzed during the current study are available from the corresponding author on reasonable request.

## Abbreviations

hrHPV: High risk human papillomavirus
E6: Early stage gene 6
DNA: Deoxyribonucleic acid
CCK8: Cell counting kit-8
FACS: Fluorescence activated cell sorter
L83V: Leucine at position 83 becomes valine, and so on
CIN: Cervical intraepithelial neoplasia
PCR: Polymerase chain reaction
HLA: Human lymphocyte antigen
FBS: Fetal bovine serum
NC: Negative control
OD: Optical density
