# Supplementary material for: HPV16 E6 gene polymorphisms and the functions of the mutation site in cervical cancer among Uygur ethnic and Han nationality women in Xinjiang, China
Source: Cancer Cell Int. 2022 Feb 22;22:94. doi: 10.1186/s12935-022-02506-0 (PMC8862000; doi:10.1186/s12935-022-02506-0)
Supplement: Supplementary file 1 — Additional file 1: Figure S1. Plasmid map of HPV16 E6. Figure S2. The expression of HPV16 E6 in C33A cells by indirect immunofluorescence (200 ×). Following the transfection of C33A cells with a GV230 empty vector, HPV16 E6 prototype vector, HPV16 E6-G295/G350 mutation vector, HPV16 E6-T295/G350 mutation vector, the red fluorescence of the vector was measured. The GV230 empty vector group was used as a control group and displayed no red fluorescence, whereas red fluorescence was observed in the other three experimental groups. The HPV16 E6-T295/G350 mutation group contained the highest fluorescence. Table S1. HPV16 E6 gene mutation loci in the non-cervical cancer of Han women. Table S2. HPV16 E6 gene mutation loci in the non-cervical cancer of Uygur women. Table S3. HPV16 E6 gene mutation loci in the cervical cancer of Han women. Table S4. HPV16 E6 gene mutation loci in the cervical cancer of Uygur women. [file 12935_2022_2506_MOESM1_ESM.docx]

**Additional file 1**

Figure S1

**
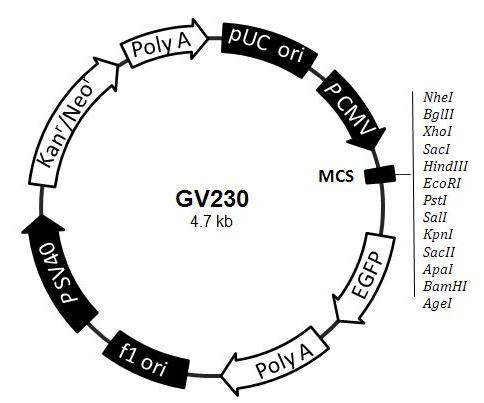
**

Figure.S1 Plasmid map of HPV16 E6

Figure S2


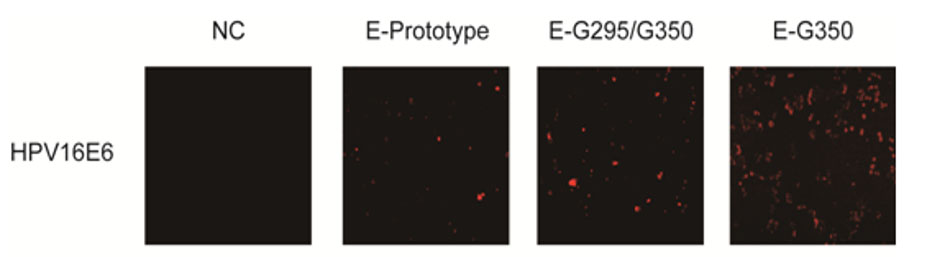


Figure S2. *The expression of HPV16 E6 in C33A cells by indirect immunofluorescence (200×).* Following the transfection of C33A cells with a GV230 empty vector, HPV16 E6 prototype vector, HPV16 E6-G295/G350 mutation vector, HPV16 E6-T295/G350 mutation vector, the red fluorescence of the vector was measured. The GV230 empty vector group was used as a control group and displayed no red fluorescence, whereas red fluorescence was observed in the other three experimental groups. The HPV16 E6-T295/G350 mutation group contained the highest fluorescence.

Table S1. HPV16 E6 gene mutation loci in the non-cervical cancer of Han women

| Mutation site | G94A | A95G | C168G | T178G | T178A | A276G | T350G | A442C |
| --- | --- | --- | --- | --- | --- | --- | --- | --- |
| Amino acid change | Syn | Syn | T22S | D25E | D25Q | N58S | L83V | E113D |
| Ep | G | A | C | T | T | A | T | A |
| AA | G | A | C | T | T | A | G | A |
| As | G | A | C | G | T | A | T | A |
| Af | G | A | C | T | T | A | T | A |
| XinJiang73 |  |  |  |  |  |  |  |  |
| XinJiang75 |  |  |  |  |  |  | G | C |
| XinJiang80 |  |  |  |  |  | G |  |  |
| XinJiang81 |  |  |  | G |  |  |  |  |
| XinJiang87 |  |  |  |  |  |  | G |  |
| XinJiang88 |  |  |  | G |  |  |  |  |
| XinJiang90 |  |  |  | G |  |  |  |  |
| XinJiang92 |  |  |  |  |  |  |  |  |
| XinJiang95 |  |  |  | G |  |  |  |  |
| XinJiang100 |  |  |  | G |  |  |  |  |
| XinJiang101 |  |  |  |  |  |  | G |  |
| XinJiang112 |  |  | G | G |  |  | G |  |
| XinJiang114 |  |  |  | G |  |  |  |  |
| XinJiang119 | a | g |  |  | A |  |  |  |
| XinJiang102 |  |  |  |  |  |  | G |  |
| XinJiang49 |  |  |  |  |  |  |  |  |
| XinJiang55 |  |  |  |  |  |  | G |  |
| XinJiang61 |  |  |  |  |  |  | G |  |
| XinJiang69 |  |  |  |  |  |  | G |  |
| total | 1 | 1 | 1 | 7 | 1 | 1 | 8 | 1 |

Table S1. EP = European standard strain; AA = Asian American; AS = Asian type; AF = African type; Syn = Synonymous. Capital letters are missense mutations, and lowercase letters are synonymous mutations.

Table S2. HPV16 E6 gene mutation loci in the non-cervical cancer of Uygur women

| Mutation site | T109C | T178G | T183G | T350G |
| --- | --- | --- | --- | --- |
| Amino acid change | Syn | D25E | I27R | L83V |
| Ep | T | T | T | T |
| AA | T | T | T | G |
| As | T | G | T | T |
| Af | T | T | T | T |
| XinJiang76 |  | G |  |  |
| XinJiang77 |  | G |  |  |
| XinJiang78 |  |  | G |  |
| XinJiang84 | c |  |  | G |
| XinJiang86 |  |  |  | G |
| XinJiang98 |  |  |  | T/G |
| XinJiang111 |  |  |  |  |
| XinJiang113 |  |  |  | G |
| XinJiang115 |  | G |  |  |
| XinJiang117 |  |  | G |  |
| XinJiang118 |  |  |  |  |
| XinJiang110 |  |  |  |  |
| XinJiang34 |  |  |  | G |
| XinJiang44 |  |  |  | T/G |
| XinJiang46 |  |  |  | G |
| total | 1 | 3 | 2 | 7 |

Table S2. EP = European standard strain; AA = Asian American; AS = Asian type; AF = African type; Syn = Synonymous. Capital letters are missense mutations, and lowercase letters are synonymous mutations.

Table S3. HPV16 E6 gene mutation loci in the cervical cancer of Han women

| Mutation site | G94A | G96A | T178G | T295G | T350G |
| --- | --- | --- | --- | --- | --- |
| Amino acid change | Syn | Syn | D25E | D64E | L83V |
| Ep | G | G | T | T | T |
| AA | G | G | T | T | G |
| As | G | G | G | T | T |
| Af | G | G | T | T | T |
| XinJiang96 |  |  |  |  |  |
| XinJiang103 |  |  | G |  |  |
| XinJiang104 |  |  |  | G | G |
| XinJiang106 |  |  |  |  |  |
| XinJiang48 |  |  | G |  |  |
| XinJiang56 |  |  |  |  | G |
| XinJiang57 |  |  |  |  |  |
| XinJiang58 |  |  |  |  | G |
| XinJiang59 |  |  |  |  | G |
| XinJiang60 |  |  | G |  |  |
| XinJiang62 |  |  |  |  | G |
| XinJiang63 |  |  |  |  |  |
| XinJiang64 | a |  |  |  | G |
| XinJiang65 |  |  |  |  | G |
| XinJiang66 |  | a |  |  | G |
| XinJiang67 |  |  |  |  |  |
| XinJiang68 |  |  |  |  | G |
| XinJiang70 |  |  |  |  |  |
| XinJiang71 | a |  |  |  | G |
| total | 2 | 1 | 3 | 1 | 10 |

Table S3. EP = European standard strain; AA = Asian American; AS = Asian type; AF = African type; Syn = Synonymous. Capital letters are missense mutations, and lowercase letters are synonymous mutations.

Table S4. HPV16 E6 gene mutation loci in the cervical cancer of Uygur women

| Mutation site | A83T | G94A | G96A | A131C | G176A | T178G | T295G | T350G |
| --- | --- | --- | --- | --- | --- | --- | --- | --- |
| Amino acid change | Syn | Syn | Syn | Syn | D25N | D25E | D64E | L83V |
| Ep | A | G | G | A | G | T | T | T |
| AA | A | G | G | A | G | T | T | G |
| As | A | G | G | A | G | G | T | T |
| Af | A | G | G | A | G | T | T | T |
| XinJiang79 |  | a |  |  |  |  |  | G |
| XinJiang83 |  |  |  |  |  | T/G |  | T/G |
| XinJiang82 |  |  |  |  |  | G |  |  |
| XinJiang91 |  |  |  |  | A |  |  |  |
| XinJiang120 |  |  |  |  |  | G |  |  |
| XinJiang121 |  |  |  |  |  | G |  |  |
| XinJiang122 |  |  |  |  |  | G |  |  |
| XinJiang1 |  |  |  |  |  |  |  | G |
| XinJiang2 |  |  |  |  |  |  |  | G |
| XinJiang3 |  |  |  |  |  |  |  | G |
| XinJiang4 |  |  |  |  |  |  |  |  |
| XinJiang5 |  |  |  | c |  | G |  |  |
| XinJiang6 |  |  |  |  |  |  |  | G |
| XinJiang7 |  |  |  |  |  |  |  | G |
| XinJiang8 |  |  | a |  |  |  |  | G |
| XinJiang9 |  |  |  |  |  |  |  | G |
| XinJiang10 |  |  |  |  |  |  |  | G |
| XinJiang11 |  |  |  |  |  |  |  | G |
| XinJiang12 |  |  |  |  |  |  |  | G |
| XinJiang13 |  |  |  |  |  |  | G | G |
| XinJiang14 |  |  |  |  |  |  |  | G |
| XinJiang15 |  |  |  |  |  |  |  | G |
| XinJiang16 |  |  |  |  |  |  | G | G |
| XinJiang17 |  |  |  |  |  |  |  | G |
| XinJiang18 |  |  |  |  |  |  |  |  |
| XinJiang19 |  |  |  |  |  |  |  | G |
| XinJiang20 |  |  |  |  |  |  | G | G |
| XinJiang21 |  |  |  |  |  |  |  |  |
| XinJiang22 |  |  |  |  |  |  |  |  |
| XinJiang23 |  |  |  |  |  |  |  | G |
| XinJiang24 |  |  |  |  |  |  | G | G |
| XinJiang25 |  |  |  |  |  |  |  |  |
| XinJiang26 |  |  |  |  |  |  |  | G |
| XinJiang27 |  |  |  |  |  |  |  | G |
| XinJiang28 |  |  |  |  |  |  |  |  |
| XinJiang29 |  |  |  |  |  |  |  | G |
| XinJiang30 |  |  |  |  |  |  | G | G |
| XinJiang31 |  |  |  |  |  |  |  | G |
| XinJiang32 |  |  |  |  |  |  |  | G |
| XinJiang33 |  |  |  |  |  |  |  |  |
| XinJiang35 |  |  |  |  |  |  |  | G |
| XinJiang36 |  |  |  |  |  |  |  |  |
| XinJiang37 |  |  |  |  |  |  |  | G |
| XinJiang38 |  |  |  |  |  |  |  | G |
| XinJiang39 |  |  |  |  |  |  |  | G |
| XinJiang40 |  |  |  |  |  |  |  | G |
| XinJiang41 |  |  |  |  |  |  |  | G |
| XinJiang42 |  |  |  |  |  |  | G | G |
| XinJiang43 |  |  |  |  |  |  |  | G |
| XinJiang45 |  |  |  |  |  |  |  |  |
| XinJiang47 | t |  |  |  |  |  |  |  |
| XinJiang50 |  |  |  |  |  |  |  | G |
| XinJiang51 |  |  |  |  |  |  |  | G |
| XinJiang52 |  |  |  |  |  |  |  | G |
| XinJiang53 |  |  |  | c |  | G |  |  |
| XinJiang54 |  |  |  |  |  |  |  | G |
| XinJiang72 |  |  |  |  |  |  |  | G |
| total | 1 | 1 | 1 | 2 | 1 | 7 | 6 | 40 |

Table S4. EP = European standard strain; AA = Asian American; AS = Asian type; AF = African type; Syn = Synonymous. Capital letters are missense mutations, and lowercase letters are synonymous mutations.
